# Dataset on stand structural indices and forest ecosystem naturalness in hemiboreal forests

**DOI:** 10.1016/j.dib.2020.105387

**Published:** 2020-03-06

**Authors:** Henn Korjus, Andres Kiviste, Ahto Kangur, Teele Paluots, Diana Laarmann, Eneli Põldveer

**Affiliations:** Chair of Forest Management Planning and Wood Processing Technologies, Estonian University of Life Sciences, Kreutzwaldi 5, Tartu, 51006, Estonia

**Keywords:** Forest assessment, Species mingling, Deadwood, Diameter differentiation, Tree positioning patterns, Scots pine, Norway spruce

## Abstract

The data paper refers to the research article “Assessment of spatial stand structure of hemiboreal conifer dominated forests according to different levels of naturalness” [1]. Forest ecosystem structure was quantified by using structural indices based on the nearest-neighborhood approach for individual trees. Species mingling, deadwood mingling, deadwood distribution, diameter differentiation and the uniform angle indices characterize the patterns of the complexity and diversity of forest ecosystems, including the arrangement of tree dimensions, species and deadwood as well as tree positioning regularities. The data is collected all over Estonia from the Estonian Network of Forest Research Plots; altogether 212 sample plots were used in this study. The plots of the Estonian Network of Forest Research Plots are re-measured in nature with an interval of five years. Forests were classified by their naturalness level as managed forests, recovering forests and natural forests. The information in this paper can be used by forest inventories for developing the methods of ecosystem naturalness assessment as well as for analysing naturalness, stand structures and tree patterns in hemiboreal forest ecosystems.

## Specifications Table

**Table:** 

Subject	Forestry
Specific subject area	Forest assessment, Forest ecology
Type of data	Table
How data were acquired	Field measurements of trees on permanent sample plots in Estonia and calculations based on these measurements. The naturalness scoring was done by scoreboard type assessment during field survey including assessment of biological values (including the presence of different stand elements and deadwood as well as deadwood decay classes), cultural-biological values and human impact to a forest stand. Individual naturalness levels were ranked according to forest site type and the naturalness score. Individual tree indices based on the nearest-neighborhood approach were calculated using the attributes associated with the tree positions, tree species and dimensions. All the indices were assessed at the single-tree level for all trees (reference tree). For assessment, all trees with DBH ≥ 4 cm were used, however understorey trees were excluded. For deadwood analysis, standing dead and broken trees were also included. The four neighbors of a reference tree were used in the assessment. Also mean index values were calculated for the plots.
Data format	Raw, analyzed
Parameters for data collection	The data is collected on forest permanent sample plots of the Estonian Network of Forest Research Plots. There are 212 sample plots and 26,931 trees in the dataset, the study included 123 plots in managed forests, 58 plots in recovering forests, and 31 plots in natural forests. The plots belonged to *Oxalis, Oxalis-Rhodococcum* and *Oxalis-Myrtillus* site types according to Lõhmus [2]. These sites were Scots pine and Norway spruce dominated mixed forests on mineral soils. Information included individual tree data: tree species, tree diameter, tree height, tree vitality status and the position of a tree.
Description of data collection	The data was collected during fieldwork, all trees were mapped and measured on forest permanent sample plots. The plots are re-measured with an interval of five years. Ecological quality of the sampled stands was evaluated using the naturalness scoring method [3] for classifying forest stands according to naturalness into managed forests, recovering forests, and natural forests.
Data source location	Institution: Chair of Forest Management Planning and Wood Processing Technologies, Estonian University of Life Sciences City: Tartu Country: Estonia
Data accessibility	Repository name: Mendeley Data Data identification number: 10.17632/t79jcv5389.2 Direct URL to data: https://data.mendeley.com/datasets/t79jcv5389/2
Related research article	Eneli Põldveer, Henn Korjus, Andres Kiviste, Ahto Kangur, Teele Paluots, Diana Laarmann. Assessment of spatial stand structure of hemiboreal conifer dominated forests according to different levels of naturalness. Ecological Indicators, 110 (2020), 105,944. DOI: https://doi.org/10.1016/j.ecolind.2019.105944

## Value of the data

• This dataset can be used to analyze naturalness, stand structures and tree patterns in hemiboreal forest ecosystems and to compare it with other boreal and temperate forests.
• This dataset will assist forest researchers to collaborate as well as to combine and extend their own data for further meta-analysis.
• This dataset can be used as training data for students in forest ecosystem modeling exercises.
• This dataset can serve as a basis for developing a method of ecosystem naturalness assessment based on stand structural indices for forest inventories.

## 1 Data

This dataset presents fieldwork data in Excel spreadsheet that was collected to analyze stand structural indices and forest naturalness in hemiboreal forest ecosystems [1]. There are three tables in the dataset: “Database information” includes description of variables and encoding, “Plots” contains plot-level data (Table 1) and “Trees” contains tree-level data (Table 2). Tree species abbreviations are presented in the Table 3.

**Table 1:** Explanation of variables in the table “Plots”.

Variable	Explanation
Plot	Number of permanent sample plot
Coordinate N	Latitude North
Coordinate E	Longitude East
Year	Year of measurement
Dominant tree species	Dominant tree species of a stand (see Table 3 for abbreviations)
Site type	Forest site type according to Lõhmus [2]
Stand age	Stand age, years
Plot radius	Plot radius, m
Naturalness score	Numeric naturalness score according to Korjus [3]
Naturalness level	Ecosystem naturalness level: B- natural stand; C - recovering stand; D - managed stand
Deadwood mingling	Mean deadwood mingling index of trees in the stand
Deadwood distribution	Mean deadwood distribution index of trees in the stand
Species mingling	Mean species mingling index of trees in the stand
Expected mingling	Mean expected species mingling index of trees in the stand
Diameter differentiation	Mean diameter differentiation index of trees in the stand
Uniform angle	Mean uniform angle index of trees in the stand

**Table 2:** Explanation of variables in the table “Trees”.

Variable	Explanation
Plot	Number of permanent sample plot
Year	Year of measurement
Tree	Number of a tree at the plot
Species	Tree species abbreviation (see Table 3)
DBH	Tree diameter at 1.3 m height
Alive	Status of tree: 1-tree is alive; 0 - tree is dead
Deadwood indices	Deadwood distribution index (if Alive = 1) or deadwood mingling index (if Alive = 0) of the tree
Species mingling	Species mingling index of the tree
Diameter differentiation	Diameter differentiation index of the tree
Uniform angle	Uniform angle index of the tree

**Table 3:** Abbreviations of tree species.

Abbreviation	Common and Latin name of tree species
HB	Common aspen (*Populus tremula*)
JA	Scots elm (*Ulmus glabra*)
KD	Common juniper (*Juniperus communis*)
KP	European white elm (*Ulmus laevis*)
KS	Silver birch (*Betula pendula*)
KU	Norway spruce (*Picea abies*)
LH	Larch (*Larix* spp.)
LM	Black alder (*Alnus glutinosa*)
LV	Gray alder (*Alnus incana*)
MA	Scots pine (*Pinus sylvestris*)
PA	Goat willow (*Salix caprea*)
PI	Rowan (*Sorbus aucuparia*)
PN	Small-leaved lime (*Tilia cordata*)
RE	Willow (*Salix* spp.)
SA	Common ash (*Fraxinus excelsior*)
SP	Common hazel (*Corylus avellana*)
TA	Common oak (*Quercus robur*)
TL	Other deciduous species
TM	Bird cherry (*Prunus padus*)
TU	Unidentified species
VA	Norway maple (*Acer platanoides*)

There are 212 sample plots and 26,931 trees in the dataset, the study included 123 plots in managed forests, 58 plots in recovering forests, and 31 plots in natural (including old-growth) forests. The plots were in Scots pine and Norway spruce dominated stands in *Oxalis, Oxalis-Rhodococcum* and *Oxalis-Myrtillus* site types. These site types are characterized usually by conifer dominated oligo-mesotrophic and mesotrophic forests on fertile mineral soils. Mean age of the studied stands was 50–153 years, mean diameter 20–39 cm, mean height 20–30 m, volume of living trees 310–510 m³ ha^−1^ and volume of deadwood 15–85 m³ ha^−1^.

Structural indices were assessed for all trees based on four nearest neighbors. Sample plot edge correction was applied to the trees near to sample plot boundary, these trees were not included in the calculations of the sample plot mean values. Updated information about the stand structural indices is available at www.pommerening.org (including R scripts).

## 2 Experimental design, materials, and methods

The measurements are done on circular plots that are located throughout Estonia and belong to the Estonian Network of Forest Research Plots [5]. Plot radius was 15–30 m depending from the tree density in the stand. All trees on the sample plots, that had diameter at breast height 4 cm or more, were measured by species, diameter at breast height, vitality status, and tree location within the sample plot. On some plots the minimum tree diameter limit was lower, 2 cm.

The forest naturalness scoring method is based on the approach of the Estonian Forest Conservation Area Project (EFCAN) [3,4] and it was used to assess individual naturalness traits of the stands where sample plots were located. EFCAN method is a scoreboard type visual evaluation of the individual naturalness traits during field survey. The scoring takes into account biological values (ecosystem legacies, deadwood quantity and properties, deadwood decay classes etc.) in the stand as well as positive and negative human impacts on the forest ecosystem using the scale: zero (no such naturalness trait in the stand), one point (only few features of an individual naturalness trait are present in the stand), two points (moderate features of an individual naturalness trait are present in the stand), and three points (with many features and strong impact of an individual naturalness trait are present in the stand). Scoring in the case of positive and negative human impacts follows the similar idea: one point – low impact to three points – strong impact. This results to an individual naturalness score for a forest plot. The plots were ranked as managed forests, recovering forests and natural forests according to naturalness score using Korjus [4] grouping methodology. Forests in this dataset growing on medium to high fertility soils were grouped to managed forest if the score was under 15 points, to recovering when the score was from 16 to 34 points, and to natural if the score was 35 points or more. Managed forests represent relatively homogeneous even-aged stands with notable signs of human activity (e.g. planted trees, stumps or drainage systems). Recovering forests are naturally or artificially regenerated, there can be visible signs of non-recent management, and there are more ecosystem legacies than in the managed forests, e.g. standing and lying deadwood and/or variable species mingling and notable age variation of trees. Natural forests originate from naturally regeneration and there are no visible signs of direct human influence, usually these are uneven-aged Multiple species stands with uneven spacing of trees, including large gaps.

The spatial forest structure was characterized by structural indices based on the nearest neighbors approach using four nearest neighbors. The indices were calculated at the single tree level for all trees, excluding underbrush. For deadwood indices, all standing dead trees and broken trees were used in calculations. Sample plot edge correction was applied to the object trees near to sample plot boundary. Such object trees may have near a neighboring tree located outside of the plot. These object trees were not included in the calculations of the mean index values of a plot.

## References

[1] Põldveer E., Korjus H., Kiviste A., Kangur A., Paluots T., Laarmann D. (2020). Assessment of spatial stand structure of hemiboreal conifer dominated forests according to different levels of naturalness. Ecol. Indic..

[2] Lõhmus E. (2004). Eesti Metsakasvukohatüübid [Estonian Forest Site Types].

[3] Viilma K., Öövel J., Tamm U., Tomson P., Amos T., Ostonen I., Sørensen P., Kuuba R. (2001). Final Report of the Estonian Forest Conservation Area Network Project.

[4] Korjus H. (2002). Inventorying natural values in forest stands. For. Stud..

[5] Kiviste A., Hordo M., Kangur A., Kardakov A., Laarmann D., Lilleleht A., Metslaid S., Sims A., Korjus H. (2015). Monitoring and modeling of forest ecosystems: The Estonian Network of Forest Research Plots. For. Stud..

